# Quality assurance in anti-tuberculosis drug procurement by the Stop TB Partnership—Global Drug Facility: Procedures, costs, time requirements, and comparison of assay and dissolution results by manufacturers and by external analysis

**DOI:** 10.1371/journal.pone.0243428

**Published:** 2020-12-03

**Authors:** Cathrin Hauk, Simon Schäfermann, Peter Martus, Nigorsulton Muzafarova, Magali Babaley, Brenda Waning, Lutz Heide

**Affiliations:** 1 Pharmaceutical Institute, Eberhard Karls University Tübingen, Tübingen, Germany; 2 Institute for Clinical Epidemiology and Applied Biometry, Eberhard Karls University Tübingen, Tübingen, Germany; 3 Global Drug Facility, Stop TB Partnership, Geneva, Switzerland; Jamia Hamdard, INDIA

## Abstract

**Background:**

Quality-assured medicines are a principal means of achieving health-related Sustainable Development Goals. An example of quality assurance/quality control (QA/QC) procedures in drug procurement is provided by the operation of the Global Drug Facility (GDF) of the Stop TB Partnership, the largest provider of tuberculosis (TB) medicines to the public sector worldwide.

**Methods:**

Procedures and results of GDF’s quality assurance/quality control (QA/QC) over the five-year period 2013–2017 were analysed retrospectively. 13,999 batches of 51 different medicines had been procured and reviewed within this period. 1,388 of these batches had been analysed in the laboratories of GDF’s external quality control agent (QCA). Assay and dissolution results determined by the manufacturers and by the external QCA were compared using Bland-Altman analysis.

**Results:**

All investigated batches of medicines were in specifications at the time of shipment. The costs for QA/QC were 0.8% of purchase costs. The median time required for chemical analysis was 10 working days. Comparison of the medicine quality analysis results showed for the poorly water-soluble compound rifampicin a bias of 4.4%, with the manufacturers reporting higher values than the external QCA, most likely due to different methods employed for the analysis. Overall 95% limits of agreement (LOAs) were -6.7 to +8.0% for assay, and -10.1 to +11.8% for dissolution. In case of kanamycin injections, 95% LOAs for assay reached -14.5 to +13.2%, largely attributable to samples from one manufacturer who had used a microbiological assay while the external QCA had used an HPLC assay.

**Conclusions:**

GDF’s procedures represent a useful benchmark when evaluating QA/QC procedures of other medicine procurement operations. Inter-laboratory comparison using Bland-Altman plots allows to investigate bias and variability in medicine quality control and should be considered as a routine procedure by drug procurement agencies, to identify priorities for further improvements.

## Introduction

Improving access to quality-assured medicines is a principal means of achieving health-related Sustainable Development Goals and Universal Health Coverage [1]. However, according to a recent WHO literature survey, poor-quality medicines constitute approximately 10% of all medicines in low- and middle-income countries [2]. The number of deaths resulting annually from the use of poor-quality anti-infective medicines is estimated as 72,000–169,000 for childhood pneumonia, and 31,000–116,000 for malaria [2]. Arguably, the most effective intervention to counter the problem of substandard and falsified medicines is to strengthen quality assurance in drug procurement. General recommendation for quality assurance in medicine procurement agencies are available [3–6]. However, the scientific literature is virtually devoid of detailed empirical data on procedures, costs and time requirements of quality assurance/quality control (QA/QC) in drug procurement. Such data are needed especially at present, since stagnating donor health funding forces many countries to expand their national procurement processes, including medicine procurement for AIDS, tuberculosis (TB) and malaria [7]. This can introduce the risk of purchasing medicines of unknown quality, and thereby also exacerbate the growing global health challenge of serious drug-resistant infections [7, 8]. Reportedly, 29 low- and middle-income countries purchased TB medicines of unknown quality between 2016 and 2018 [8]. National procurement agencies, regulatory authorities and non-governmental organizations therefore need information how to further improve quality assurance in drug procurement. The present study attempts to report such information.

Quality-assured medicines are of particular importance in the case of anti-TB medicines since poor quality anti-TB medicines are among the drivers of the emergence of drug-resistant TB pathogens [9–12]. Tuberculosis is the ninth leading cause of death worldwide, and is the leading cause of death from a single infectious agent, ranking above HIV/AIDS [13]. In drug-susceptible TB, a six-months regimen involving four first-line drugs (rifampicin, isoniazid, ethambutol and pyrazinamide) achieves treatment success rates of at least 85% [13]. The required medicines could be purchased for only 27 US$ per treatment course from the Global Drug Facility in 2019 [14]. However, drug-resistant TB is a continuing threat, with 490,000 cases of multi-drug resistant TB (MDR-TB) reported in 2017 [13]. MDR-TB must be treated with second-line anti-TB drugs, and they currently cost approximately 485–1850 US$ per treatment course [14], i.e. 20–70 times more than first-line treatments.

The Global Drug Facility (GDF) of the Stop TB Partnership was founded in 2001, in order to ensure uninterrupted access to quality-assured anti-TB medicines. Today, GDF is the world’s largest provider of TB products for national TB control programs. The Global Fund to Fight AIDS, Tuberculosis and Malaria (GF), the United States Agency for International Development (USAID), as well as governments and other non-governmental organizations purchase anti-TB medicines and diagnostics from GDF for TB control programs especially in developing countries. Both donor funding and national government funding is used for these purchases. In 2017, GDF delivered TB medicines and diagnostics of a total value of 304 million US$ to 119 countries. GDF pursues active market shaping policies to optimize price, quality and sustainable supply of TB products, and also offers support and technical advice to national TB programs and policy makers. GDF’s policies, market shares and its influence on price of TB products have been analysed in previous studies [15–17].

At least for essential medicines procured with donor funds, it has been suggested that results from quality assurance should be shared among different stakeholders [4]. Likewise, GDF’s “Quality Assurance Policy and Procedures” state the aim to share information on quality aspects of medicines with other major international organisation and donors [18]. Yet, to the best of our knowledge there is no published study in the scientific literature which reports quantitative analytical results of medicine quality testing in drug procurement.

Therefore, the present study was carried out with two aims:

1. We report on the procedures, costs and time requirements of GDF’s medicine quality assurance/quality control operation in the five-year period of 2013–2017. During this period, GDF procured 13,999 batches of medicines, and GDF’s external quality control agent selected 1,388 of these batches for analysis in its WHO-prequalified medicine quality control laboratories.
2. We carried out an inter-laboratory comparison of the analytical results provided by GDF’s commercial drug suppliers and GDF’s external quality control agent (QCA). The results suggest that such comparisons are useful to guide further improvements of the QA/QC efforts in drug procurement and should be considered as a routine procedure in drug procurement organizations.

## Methods

### Data sources

In the procurement of anti-TB medicines by GDF, the IDA Foundation, Amsterdam, NL, has been contracted to provide procurement services for anti-TB medicines. Through this agency, SGS Netherlands B.V. (Spijkenisse, NL) has been subcontracted as external quality control agent (QCA), to provide quality control services. For the present study, the QCA provided Microsoft Excel files on all 13,999 batches of medicines procured and reviewed in the five-year study period 2013–2017, as well as pdf files with the results of external analysis carried out on 1,388 of these batches in the WHO-prequalified laboratories of the QCA in India and in Belgium. Since 2015, the QCA had furthermore carried out Critical CoA Reviews (see Results section), and GDF provided all customer complaints received in the period 2015–2017.

The QCA had routinely entered the data of the manufacturers’ Certificates of Analysis (CoAs), including quantitative results on assay and dissolution, into Excel files. In contrast, the data of the medicine quality analyses in the QCA’s laboratories had not been entered into a database prior to this study and were provided by the QCA in form of pdf files. For the present study, assay and dissolution results for selected samples (see below) were manually transferred from these pdf files into a single Excel data file. Additionally, the mean of the dissolution values of the six units investigated in S1 stage was calculated. Correct transfer was checked by two independent investigators.

### Random selection of 196 medicine batches for inter-laboratory comparison of assay and dissolution results from manufacturer analysis and from external QCA laboratory analysis

Out of the 1,388 batches which had been analysed in the laboratories of the QCA in the study period 2013–2017, 196 were selected within the present study for a retrospective inter-laboratory comparison of assay and dissolution results. The 1,388 batches were sorted into strata according to manufacturers and Finished Pharmaceutical Products (FPPs). This resulted in 69 strata containing between 1 and 161 batches (median 10 batches). Random numbers were assigned to each batch using the RND function of Microsoft Excel, and batches were selected based on highest random numbers per stratum. According to the size of the respective stratum, different numbers of batches were selected: if a stratum contained 1–5; 6–25; 26–125; >125 batches, then 1; 2; 3; or 4 batches were selected, respectively. However, a minimum of five batches for each active pharmaceutical ingredient (API) from each manufacturer was selected if possible; if less than five batches for a given API from a given manufacturer had been analysed by the QCA in the study period, all analysed batches were included. This resulted in the selection of 196 batches for inter-laboratory comparison of assay and dissolution results.

### Statistical analysis

Statistical analysis included the comparison of assay and dissolution results reported by the manufacturers and reported by the external QCA. The primary analysis was done graphically using ordinary scatterplots and Bland–Altman plots including limits of agreement [19]. Additionally, t-tests were done to examine systematic differences, and Spearman correlations were calculated. Analysis of variance was applied to compare results between APIs and manufacturers. In these analyses only groups with at least 15 samples were included. The level of significance was 0.05 (two-sided) in all statistical tests. Exact two-sided 95% confidence limits for proportions were reported. As the primary analysis was descriptive, no adjustment for multiple testing was applied. All analyses were done using SPSS for Windows release 24, exact confidence limits were obtained using the binom.test procedure in R release 3.2.2.

## Results

### Overview of products, their prequalification status and their manufacturers

During the study period 2013–2017, GDF’s external Quality Control Agent (QCA) monitored quality data of 51 different medicines, representing 26 active pharmaceutical ingredients (APIs) in different formulations, dosages and fixed-dose-combinations. A list of these medicines, including the estimated annual quantities procured for each product [20, 21], is given in S1 Table. In total, quality data of 13,999 batches of medicines was monitored, including 6957 batches of first-line adult medicines, 852 batches of first-line paediatric medicines and 6190 batches of second-line medicines.

S1 Scheme summarizes the principles of GDF’s supplier and product selection procedure. Any Finished Pharmaceutical Product (FPP) procured through GDF must either be prequalified within the WHO Prequalification of Medicines Programme (WHO PQP) [22], or approved by a Stringent Regulatory Authority (SRA) [18, 23]. When only one or no product with WHO prequalification or SRA approval is available on the global market, GDF may procure External Review Panel (ERP)-recommended products for a period of up to 12 months. In the beginning of GDF’s operations, the progress of the prequalification of anti-TB medicines was still limited [24]. Meanwhile, however, progress is remarkable: of all 13,999 medicine batches reviewed in the study period 2013–2017, 69.4% represented WHO-prequalified products, 24.4% SRA-approved products, and 6.2% ERP-approved products. An ongoing further shift from ERP-recommended to SRA-approved or WHO-prequalified products is noticeable: as of January 2018, only two medicines on the GDF medicines list were ERP-recommended, all the others were either WHO-prequalified, or SRA-approved (S1 Table). From the data in S1 Table, it can be estimated that at present 87% of all medicine batches procured for GDF represent WHO-prequalified products, 13% SRA-approved products, and only 0.2% ERP-recommended products. Among the first-line adult medicines, WHO-prequalified products constitute even 97%.

In order to ensure continuous supply and cost-effective procurement, GDF aims to contract more than one supplier for each product [18, 25]. As shown in S1 Table, within the study period 32 of the 51 provided medicines were procured from two or more manufacturers, and these products represented 91% of the reviewed batches. The most notable product which still had to be procured from only one single source was streptomycin injections.

**Fig 1:**
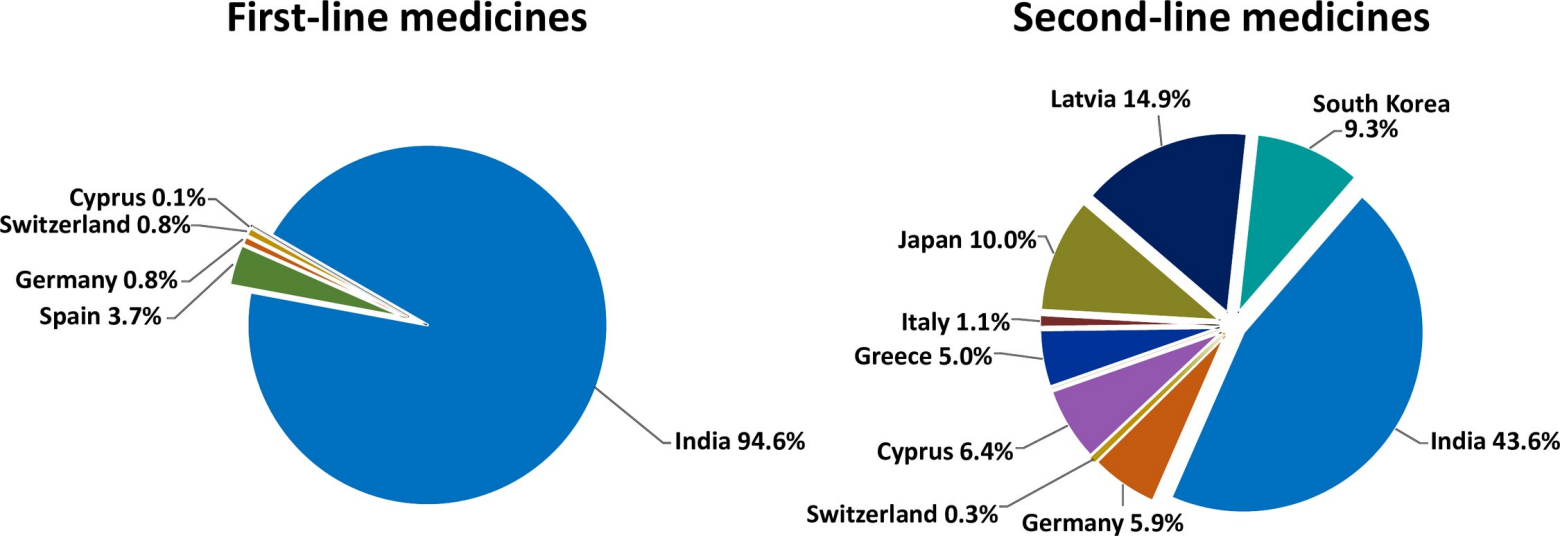
Origin of anti-tuberculosis medicines procured by the Global Drug Facility. Percentages are based on the number of batches procured in the years 2013–2017. Information on the number of units is given in S1 Table.

Within the study period, GDF procured its medicines from a total of 33 manufacturers. Fig 1 shows the predominance of manufacturers from India for the supply of first-line anti-TB medicines, whereas second-line medicines come from a wider range of countries.

### Procedures and methods for quality control

The principles of GDF’s multi-step quality control procedure are shown in S2 Scheme. It comprises “Critical CoA Reviews” which are generated by GDF’s Quality Control Agent (QCA) when a product is procured for the first time from a given manufacturer, and which reviews the appropriateness of the manufacturers’ analytical methods and specifications. Thereafter, the QCA performs routine “CoA Reviews”, i.e. reviews of the Certificates of Analysis provided by the manufacturers for every batch. This resulted in 13,999 CoA reviews prepared by the QCA in the study period 2013–2017. In 303 cases, this reviewing of the manufacturers’ CoAs showed the need for clarifications or corrections by the manufacturer. The median time required for these clarifications was four days, although some cases required much longer time (range 1–113 days, mean 20 days).

Using a risk-based random selection process, GDF’s QCA selected 1,388 batches in the study period for laboratory analysis in its own laboratories. These carry out analytical tests as listed in S2 Scheme. Whenever possible the methods of analysis of the International Pharmacopoeia (Ph. Int.), the British Pharmacopoeia (BP) and the United States Pharmacopeia (USP) are followed. However, Ph. Int. monographs exist for only 27 of the 51 products listed in S1 Table, and for six products no monographs exist at all in any of the three named pharmacopeias. Even when compendial methods exist, the manufacturer may follow his own in-house methods for analysis, e.g. since at the time of SRA/ERP approval or WHO prequalification of the product the manufacturer may have used and documented that in-house method. In such cases, it is sometimes necessary that the QCA establishes and validates the manufacturers’ in-house method in his own laboratory (“method transfer”), a particularly time- and finance-consuming process. In the study period 2013–2017, such method transfers had to be carried out in 17 cases.

### Medicines identified as out-of-specification

Within the study period, out of the 1,388 batches tested by GDF’s external QCA following the above procedure, not a single batch was found to be out-of-specification at the time of shipment (upper 95% confidence limit 0.27%). However, 15 customer complaints were reported to GDF in the period 2015–2017, claiming quality problems observed after receipt of the shipment by the customers. In five of these cases, the products were indeed confirmed to be out-of-specifications, and these batches were replaced by the manufacturers upon request of GDF. Four of these five cases concerned pyridoxine tablets that showed discoloration; notably, three of these cases concerned the same batch. The fifth of these cases represented improperly sealed sachets of *para*-aminosalicylic acid, leading to swelling of the sachets. In one further case, the sample in question showed colour changes and the presence of degradation products which was most likely caused by inadequate storage temperature. In another case, the sample was expired. In four further cases, chemical analysis proved that the products were compliant with specifications. The four final cases were inconclusive as insufficient information or sample material was provided with the complaint.

### Cost and time requirements of quality control measures

During most of the study period, GDF charged customers 1.2% of the purchase costs of the consignments for medicine quality analysis. Starting from June 2017, the charges were reduced to 0.8%. We estimated that laboratory analyses (of 1,388 batches) accounted for 64% of the quality control costs, while review of the manufacturer CoAs (of 13,999 batches) accounted for 29%, and other services for 7%.

The time from the random selection of batches for laboratory analysis to the conclusion of laboratory results (i.e. the time for sample collection, forwarding of samples to the laboratory, and analysis) was remarkably short. The QCA records showed that for 47% of the investigated batches, the lab result became available in the same calendar month in which it had been requested, and for 46% in the following calendar month. This process took longer only in 7% of the cases. The median time for analysis alone (i.e. from arrival of the samples in the QCA’s laboratory until availability of the results in the QCA’s Netherland office) was only 10 working days (mean: 11 working days; range: 0–64 working days).

### Inter-laboratory comparison of assay and dissolution results from manufacturer analysis and from external QCA laboratory analysis

For inter-laboratory comparison of assay and dissolution results from manufacturer analysis and from external QC laboratory analysis, 196 of the 1,388 CoAs prepared by the QCA’s quality control laboratory were selected using a stratified random selection procedure (see Methods). The focus of this analysis was not statistical testing of a hypothesis but an estimation of limits of agreement of assay and dissolution results between manufacturer analysis and external QC laboratory analysis.

Many of the selected medicines were fixed-dose combinations containing several APIs, and both solid oral formulations and injectable formulations were included. Therefore, the selected 196 samples represented 288 assay results and 261 dissolution results, each of them with a value reported by the manufacturer and another value reported by the QCA laboratory. A descriptive summary of these data is given in Table 1. In addition, S2 Table shows descriptive summaries for each of the 13 APIs included in this analysis.

**Table 1:** Descriptive summary of assay and dissolution data included into the inter-laboratory comparison of assay and dissolution results from manufacturer analysis and from external QCA laboratory analysis.

		Assay		Dissolution		Difference	
		Manufacturer analysis	External QCA analysis	Manufacturer analysis	External QCA analysis	Assay	Dissolution
N		288	288	262	262	288	261^a^
Mean		99.8%	99.1%	98.5%	97.6%	0.67%	0.88%
Median		99.6%	99.3%	99.0%	98.3%	0.42%	0.62%
Standard deviation		1.86%	3.31%	3.55%	4.75%	3.74%	5.55%
Minimum		95.0%	90.0%	79.5%	82.0%	-15.20%	-16.65%
Maximum		108.2%	113.1%	107.5%	110.0%	15.30%	21.00%
Percentile	2.5	95.0%	92.8%	88.5%	86.7%	-6.85%	-11.52%
	25	98.7%	96.9%	97.0%	95.0%	-1.50%	-2.19%
	75	100.7%	100.7%	100.7%	100.5%	2.87%	3.50%
	97.5	103.7%	106.0%	103.6%	107.6%	8.70%	13.08%
Skewness		0.66	0.58	-1.29	-0.44	-0.31	0.22
Kurtosis		2.37	2.10	4.10	0.57	3.06	1.40

^a^ Two dissolution results (one from manufacturer analysis, one from external QCA analysis) were unavailable for the comparison.

Analysis of skewness showed that data were symmetrically distributed. Even though kurtosis exceeded the range of -1 to +1, parametrical procedures were chosen due the well-known robustness of t-tests and one-factorial ANOVAs (Table 1).

**Fig 2:**
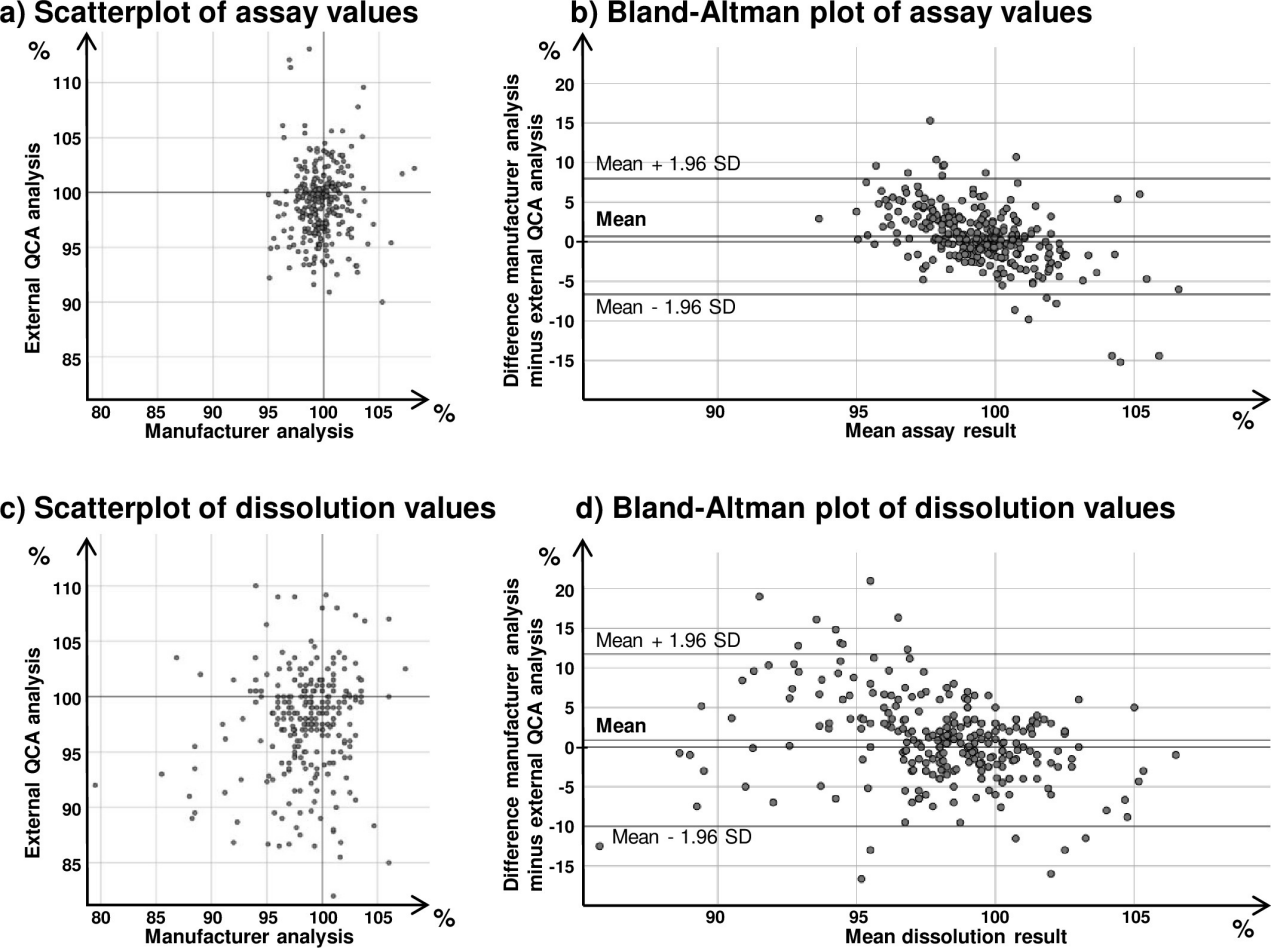
Inter-laboratory comparison of assay and dissolution results from manufacturer analysis and from external QCA laboratory analysis. Between the results of manufacturer analysis and external QCA analysis, correlation was calculated as r = 0.035 (p = 0.559) for assay results and as r = 0.132 (p = 0.034) for dissolution results. The bias depicted in the Bland-Altman plots was 0.67% for assay (two-sided t-test: p = 0.003) and 0.88% for dissolution (two-sided t-test: p = 0.011). Further results are shown in Table 2.

Fig 2A and 2C show scatterplots of the agreement of manufacturer analysis and external QCA analysis for assay and dissolution of the API. Over the narrow range of outcomes (95% of the assay results were between 93 and 106% of the declared content), correlation between the values reported by the compared laboratories was weak (see legend of Fig 2).

**Table 2:** Inter-laboratory comparison of assay and dissolution results from manufacturer analysis and from external QCA laboratory analysis: Results of Bland-Altman analysis.

Data set compared (Manufacturer analysis minus external QCA analysis)	n	Difference Mean	Limits of agreement
Assay	288	0.67%**	- 6.69% to + 8.03%
Dissolution	261	0.88%*	- 10.05% to + 11.81%
Isoniazid assay	57	1.60%**	- 5.49% to + 8.69%
Ethambutol assay	37	0.71%	- 7.10% to + 8.52%
Pyrazinamide assay	37	0.77%*	- 3.21% to + 4.75%
Rifampicin assay	51	1.30%*	- 5.93% to + 8.53%
Isoniazid dissolution	57	0.67%	- 9.91% to + 11.25%
Ethambutol dissolution	36	0.04%	- 14.08% to + 14.16%
Pyrazinamide dissolution	36	1.00%	- 6.75% to + 8.75%
Rifampicin dissolution	51	4.39%**	- 9.85% to + 18.63%
Kanamycin assay	81 ^a^	-0.65%	- 14.52% to + 13.22%
Kanamycin assay, manufacturer 1	55 ^a^	-0.19%	- 15.03% to + 14.65%
Kanamycin assay, manufacturer 2	16 ^a^	1.39%	- 1.55% to 4.33%

The highest observed bias is highlighted in bold print. Detailed results, including standard deviation, 95% confidence intervals for mean and limits of agreement, and correlation coefficients, are given in S3 Table.

* Difference is significant with p < 0.05 (two-tailed).

** Difference is significant with p < 0.01 (two-tailed).

^a^ Originally 15 kanamycin injection samples had been selected for the inter-laboratory comparison of results; a summary of these data is included in S2 Table. For the analysis shown in the last three lines of this table, the data of all 81 kanamycin injection samples which had been analysed in the study period by the external QCA were investigated; a summary of these data is given in S4 Table.

The results of Bland-Altman analysis are summarized in Table 2. A small bias was observed for both assay (0.67%) and dissolution (0.88%). As already obvious from the scatterplots, Bland-Altman analysis showed considerable random variation. Upper and lower 95% limits of agreement were calculated as -6.7 to 8.0% for assay, and -10.1 to 11.8% for dissolution. The pattern of relationship between difference and mean in Bland-Altman analysis for both assay and dissolution showed some heteroscedasticity, since values reported by the manufacturers showed less variation than values reported by the external QCA. However, over the investigated range of results this effect was small.

S1 and S2 Figs show separate scatterplots and Bland-Altman plots of assay and dissolution values for the four principal first-line anti-TB agents isoniazid, ethambutol, pyrazinamide and rifampicin. The results of the Bland-Altman analysis for these four APIs are included in Table 2. Consistent with the observation in the overall analysis shown in Fig 2, the assay values show a small bias which reaches statistical significance in case of isoniazid, pyrazinamide and rifampicin. Dissolution values show no statistically significant bias for isoniazid, ethambutol and pyrazinamide. In contrast, data for rifampicin show a bias of 4.4% (two-sided t-test p < 0.001). An analysis of variance was carried out for the rifampicin dissolution differences using manufacturer and product as factors, but showed no significant influence of these factors on the observed differences.

**Fig 3:**
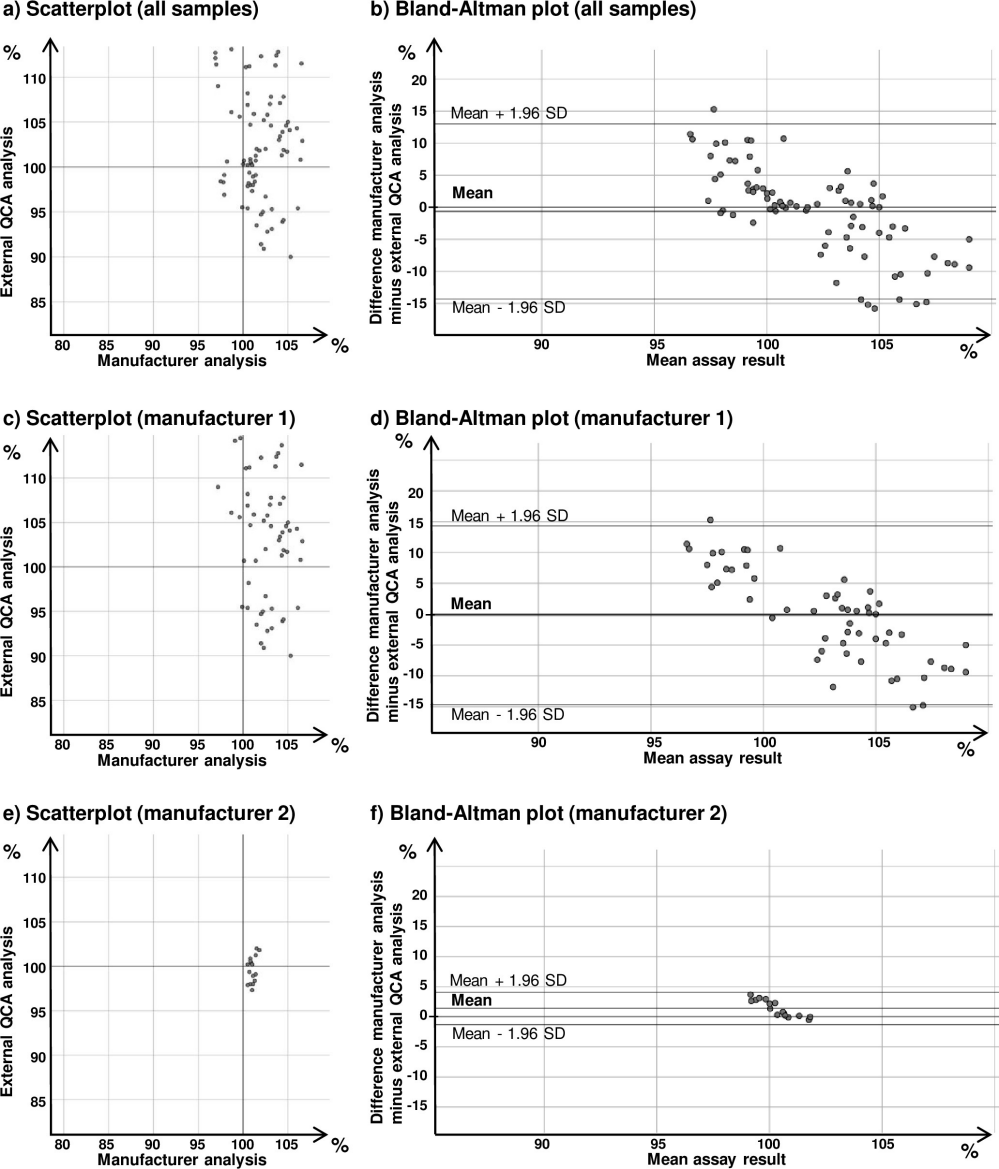
Inter-laboratory comparison of kanamycin injection assay results from manufacturer analysis and from external QCA laboratory analysis. See S4 Table for a descriptive summary of the data.

In the Bland-Altman plot in Fig 2B, six of the investigated samples show assay differences of > 10% between manufacturer analysis and external QCA analysis. Notably, five of these represented kanamycin injection samples. This prompted us to investigate the data of all 81 kanamycin samples which had been analysed in the study period by the external QCA laboratory. The assay data were manually transferred from the different files provided by the external QCA into a single data file. S4 Table shows a descriptive summary of the assay data on kanamycin injection samples. Fig 3A and 3B show the scatterplot and Bland-Altman plot for these data, and the results of Bland-Altman analysis are shown in Table 2. Upper and lower 95% limits of agreement are -14.5 to +13.2% for kanamycin assay, considerably higher as in the overall analysis of all APIs. In this case, ANOVA showed a clear influence of manufacturer. This is illustrated in the scatterplots and the Bland-Altman plots for the two most important manufacturers of kanamycin injections, which together supplied 71 of all 81 investigated kanamycin samples (Fig 3C, 3D, 3E and 3F). Manufacturer 1 (55 samples) had used a microbiological assay for kanamycin while the external QCA had used the HPLC assay of the United States Pharmacopeia; Bland-Altman analysis showed limits of agreement of -15.0 to +14.7%. In case of manufacturer 2 (16 samples), both manufacturer and the external QCA had used a microbiological assay; Bland-Altman analysis showed much narrower limits of agreement, i.e. -1.6 to +4.3%.

## Discussion

In the study period, not a single batch was found to be out-of-specifications at the time of shipment. Also, the number of customer complaints was minimal, with only three batches confirmed to be out-of-specifications (two batches of pyridoxine tablets showing discolorations, one batch of *para*-aminosalicylic acid with improperly sealed sachets). Obviously, this success was achieved not only by quality control (QC) measures (i.e. pre-shipment inspections and laboratory analyses) but by a comprehensive system of quality assurance (QA). GDF’s QA/QC procedures documented here present a successful example of quality assurance in drug procurement, evidenced both by virtual absence of out-of-specification batches and by very low requirements of time and costs. Drug procurement agencies both within and outside the field of anti-TB medicines may compare their procedures, including time and cost requirements, as well as their outcomes, including number of out-of-specification batches, to the results presented in this paper, to assess and further improve the efficiency of their operations.

Especially in the field of anti-TB medicines, impairments of their quality, as well as interruptions in their supply, may lead to an increase of cases of multi-drug resistant tuberculosis (MDR-TB) [11, 12] which thereafter can only be treated at significantly higher costs. Governments, national TB programs, technical and funding partners must ensure the continuous supply of quality assured TB medicines to avoid the amplification of multi-drug resistance of TB mycobacteria, to maintain the gains achieved so far globally and contain the cost of treatment. Therefore, while in future medicine financing is expected to shift increasingly from donor support to national funding schemes [7], it should be assured that procurement of anti-TB medicines, including QA/QC, is carried out effectively and on a sufficiently large scale.

Within the present study, an inter-laboratory comparison of the assay and dissolution values reported by the manufacturers and by GDF’s external quality control agent (QCA) was conducted. The results need to be viewed in comparison to the pass/fail thresholds given in the relevant pharmacopeial monograph. For rifampicin capsules, as example, USP 2018 states the acceptable limits for assay (= content of API) as 90–110% and for dissolution as > 75% of the declared content. Results of the inter-laboratory comparison can furthermore be viewed in comparison to the “acceptable difference” between manufacturer and external QCA analysis which is defined in a contract formulated by GDF and which specifies procedures for quality control testing by the external QCA. That contract specifies that results obtained for the tested batches by the external QCA must not differ more than 2% from the results provided by the manufacturer in case of analyses for which a manufacturer’s method has been transferred to the external QCA laboratory. As explained above, methods transfers are only conducted for a small part of the conducted analyses, and larger differences than 2% may be expected when external QCA and manufacturer use different methods.

Overall evaluation of the results for all 196 samples included into this comparison (Fig 2 and Table 2) showed a small systematic bias, with the manufacturers reporting higher, i.e. more favourable results than the external QCA. However, these biases amounted only to 0.67% (assay) and 0.88% (dissolution) of the declared content and were therefore not relevant in comparison to the pass/fail thresholds of the pharmacopeial monographs. Furthermore, it must be pointed out that a large random variation of differences may obscure systematic differences, whereas a small random variation may show differences which are significant but not relevant.

A separate analysis of the dissolution results for the four principle first-line anti-TB agents showed that the manufacturer and external QCA results were similar for the highly water-soluble compounds isoniazid, ethambutol and pyrazinamide. However, for the poorly water-soluble compound rifampicin the dissolution values stated by the manufacturer (mean = 98.5%) were clearly higher than those given by the external QCA (mean = 94.1%; bias 4.4%; two-sided t-test: p < 0.001). Dissolution testing of rifampicin is known to be problematic [26, 27], and the dissolution testing methods currently given in USP 2018 and Ph. Int. 2017 are remarkably different (S5 Table). The reasons have been reviewed by Becker et al. [28]. Rifampicin exists in different polymorphic forms of different solubility. It is highly soluble in acidic aqueous solutions but decomposes rapidly under acid conditions. The influence of this decomposition is often not considered, and therefore the wisdom of the USP 2018 method for testing the dissolution of rifampicin capsules and of rifampicin/isoniazid capsules, in 0.1 N HCl (S5 Table) has been questioned [28]. At neutral pH, solubility of rifampicin is much lower and dissolution experiments often give erratic results. These have been attributed to poor wettability of rifampicin [28]. The dissolution testing procedure of Ph. Int. 2017, carried out at pH 6.8, therefore includes sodium dodecyl sulfate as detergent (S5 Table). In several monographs for fixed-dose combinations, Ph. Int. states that methods for rifampicin dissolution testing still have to be formulated (S5 Table), forcing pharmaceutical laboratories to use in-house methods for rifampicin dissolution testing. The observed differences between manufacturer results and external QCA results for rifampicin dissolution are therefore most likely attributable to different methods employed. Notably, for all rifampicin samples investigated, the dissolution values determined by the external QCA (mean = 94.1%) were well above the pharmacopeial pass/fail thresholds (> 75% or > 80%, see S5 Table) and more conservative than the values reported by the manufacturers (mean = 98.5%). Therefore, the observed bias did not affect pass/fail decisions or patient safety. Nevertheless, the further development and harmonization of rifampicin dissolution testing methods may represent a priority for the national pharmacopeial conventions as well as for the WHO Expert Committee on Specifications for Pharmaceutical Preparations which is responsible for approving the monographs of the International Pharmacopoeia [3].

While bias (i.e. systematic difference) between manufacturer and external QCA analysis was low for most APIs, random variability was considerable. Overall 95% limits of agreement (LOAs) were calculated as -6.7 to +8.0% for assay, and -10.1 to +11.8% for dissolution (Table 2). This compared favourably with the LOAs reported in four earlier inter-laboratory comparisons in medicine quality analysis [29–32], reporting 95% LOAs of assay values of -19 to +24%; -15 to +18%; -20 to + 20%; and -13 to +20%, respectively (the latter values are calculated from the assay values of CENQAM and DCQL-Sana’a laboratories in ref. [32]). Nevertheless, the random differences observed in the present study are clearly higher than the ± 2% difference which is considered acceptable in cases when both laboratories use the same analytical method (see above). No true results for the individual analyses included in the present comparison are known, and therefore it cannot be decided to which extent manufacturer analysis and external QCA analysis contributed to the observed random variability between their results. Given that for most investigated medicines assay and dissolution results were close to 100% of the declared content (Table 1), the observed random variability will in most cases have no influence on the validity of the pass/fail classification according to the pharmacopeial limits. An exception may be presented by the investigated kanamycin assay values, where variability was especially high, and LOAs for assay were calculated as -14.5 to +13.2% (Table 2 and Fig 3). Given the USP 2018 threshold of 90–115% for kanamycin injection assay values, such wide LOAs may question the validity of the pass/fail classification of the analysis and call for careful validation of the analytical procedures both in the manufacturer’s laboratory and in the external QCA laboratory. GDF’s external QCA had already responded to this problem by arranging that all analyses for kanamycin were carried out in its especially well-experienced laboratory in Belgium. As mentioned in the results section, the high variability between manufacturer and external QCA results in kanamycin assay results could largely be attributed to samples from one manufacturer who used a microbiological assay for kanamycin while the external QCA had used the HPLC assay of the USP (Fig 3). This strikingly demonstrates the important influence of different analytical methods used. The variability of kanamycin assay results between manufacturer and external QCA analysis can apparently be resolved by method transfer, but the trueness of the results obtained with different methods should still be investigated.

## Conclusions and recommendations

1. GDF’s QA/QC procedures have proven to represent a very successful model to ensure uninterrupted access to quality-assured medicines at low prices. These procedures should be used as a benchmark when evaluating and improving QA/QC procedures of other medicine procurement operations, both within and beyond the area of anti-TB medicines.

2. GDF’s QA/QC procedures have proven to achieve complete absence of out-of-specification batches at the time of procurement. Obviously, quality of anti-TB medicines may deteriorate over time, especially (but not only) in case of inappropriate transport and storage conditions, and the stability of the medicines is dependent on the quality of formulation and packaging. Future investigations of the quality of anti-TB medicines obtained from GDF should therefore include the quality at the time of administration to the patient, in addition to quality at the time of procurement.

3. The inter-laboratory comparison carried out within this study showed that certain problems in quality analysis results, such as a bias in rifampicin dissolution values and high variability in kanamycin assay values, can be rapidly detected by systematic comparison of the results of manufacturer and external QCA analysis, e.g. during a semi-annual or annual review of the reported results. Therefore, based on the results of this study GDF has initiated steps to ensure that in future all results of manufacturer analysis and external QCA analysis are entered, by the manufacturers and/or by the external QCA, into a database of uniform format, and are evaluated routinely for criteria such as bias and limits of agreement, both for different APIs and for different manufacturers and products. This may also establish whether a given product demonstrates changing characteristics over time, e.g. undergoes significant dissolution changes compared to the original batch, which may indicate the need for the manufacturer to re-demonstrate the bioequivalence of the product.

4. Also other publicly funded procurement agencies may likewise collect and evaluate results of both manufacturer and external QCA analysis, and share results. This may facilitate the identification and dissemination of best practices in medicine QA/QC and allow to further improve procedures, e.g. with the aim to reduce the random variability observed in this study.

5. The WHO Expert Committee on Specifications for Pharmaceutical Preparations may take notice of the results of such inter-laboratory comparisons in pharmaceutical analysis and use them to identify priorities in the further development of norms, standards and guidelines, including monographs of the International Pharmacopoeia.

## Supporting information

S1 Scheme Supplier and product selection procedure of the Global Drug Facility (GDF). This scheme summarizes only the most basic principles; details are described in the references [18,25].
(PDF)

S2 Scheme Principles of the quality control procedures of the Global Drug Facility (GDF). (PDF)

S1 Fig Inter-laboratory comparison of assay results from manufacturer analysis and from external QCA laboratory analysis for the four principal first-line anti-TB agents. (PDF)

S2 Fig Inter-laboratory comparison of dissolution results from manufacturer analysis and from external QCA laboratory analysis for the four principal first-line anti-TB agents. (PDF)

S1 Table Details of anti-tuberculosis medicines monitored in the quality control procedure of the Global Drug Facility in the study period 2013–2017. (PDF)

S2 Table Descriptive summary of assay and dissolution data for all 13 active pharmaceutical ingredients which had been analysed in the study period by the external QCA. (PDF)

S3 Table Detailed results of Bland-Altman analysis. (PDF)

S4 Table Descriptive summary of assay data for all 81 kanamycin injection samples which had been analysed in the study period by the external QCA. In addition, data from the two main manufacturers supplying kanamycin are presented.
(PDF)

S5 Table Dissolution testing conditions for solid oral formulations containing rifampicin in the United States Pharmacopeia 2018 and the International Pharmacopoeia 2017. (PDF)
