# Supplementary material for: Quality assurance in anti-tuberculosis drug procurement by the Stop TB Partnership—Global Drug Facility: Procedures, costs, time requirements, and comparison of assay and dissolution results by manufacturers and by external analysis
Source: PLoS One. 2020 Dec 3;15(12):e0243428. doi: 10.1371/journal.pone.0243428 (PMC7714355; doi:10.1371/journal.pone.0243428)
Supplement: S1 Scheme — This scheme summarizes only the most basic principles; details are described in the references [18,25]. (PDF) [file pone.0243428.s001.pdf]

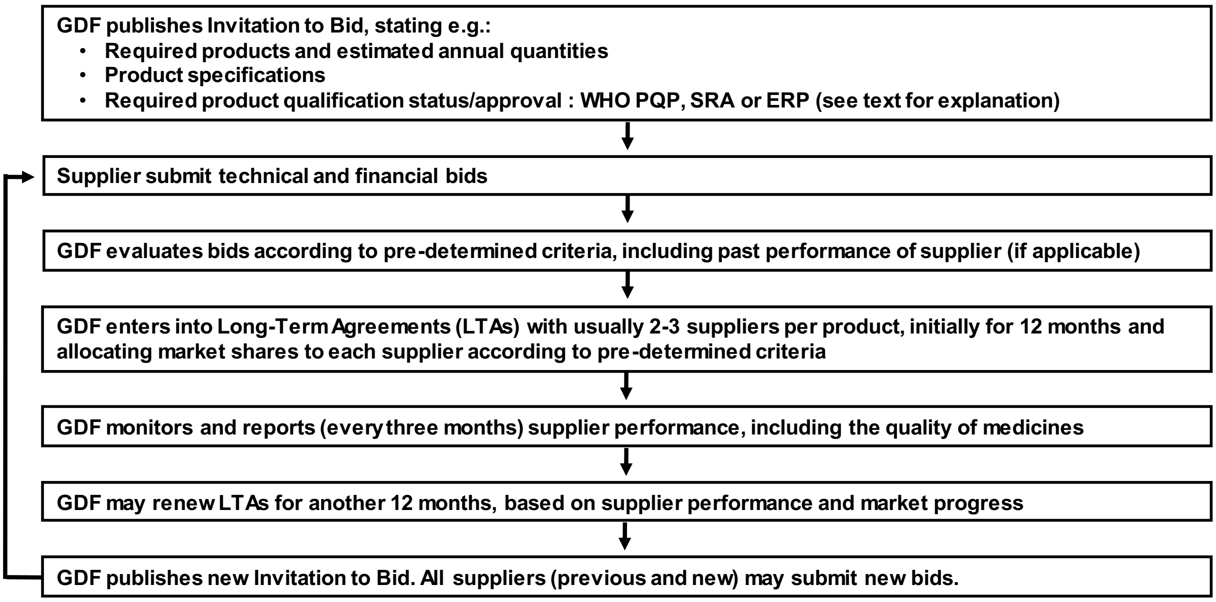

S1 Scheme. Supplier and product selection procedure of the Global Drug Facility (GDF).

This scheme summarizes only the most basic principles; details are described in the references [18,25].
