# Supplementary figures and images for: Quality assurance in anti-tuberculosis drug procurement by the Stop TB Partnership—Global Drug Facility: Procedures, costs, time requirements, and comparison of assay and dissolution results by manufacturers and by external analysis
Source: PLoS One. 2020 Dec 3;15(12):e0243428. doi: 10.1371/journal.pone.0243428 (PMC7714355; doi:10.1371/journal.pone.0243428)

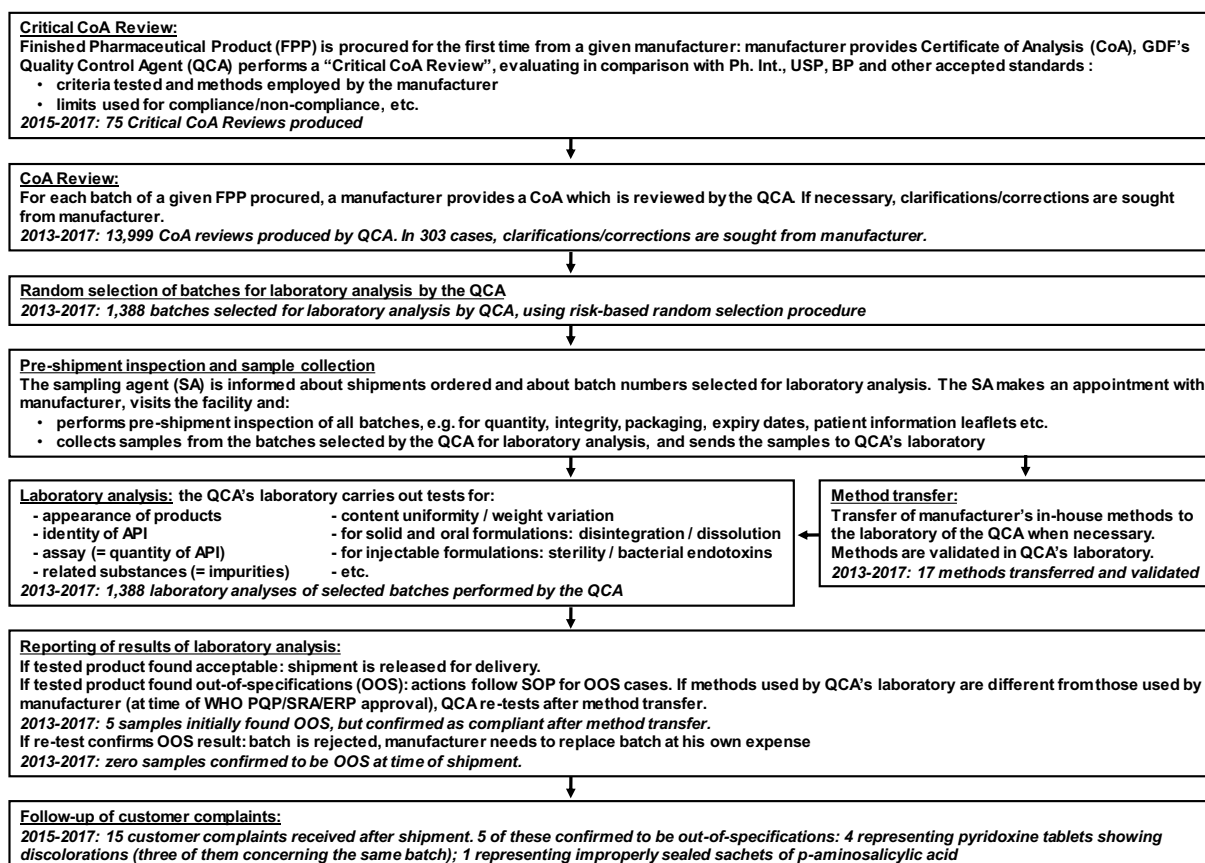

**S2 Scheme. Principles of the quality control procedures of the Global Drug Facility (GDF).**

Supplement: S2 Scheme — (PDF) [file pone.0243428.s002.pdf]
