# Supplementary material for: Quality assurance in anti-tuberculosis drug procurement by the Stop TB Partnership—Global Drug Facility: Procedures, costs, time requirements, and comparison of assay and dissolution results by manufacturers and by external analysis
Source: PLoS One. 2020 Dec 3;15(12):e0243428. doi: 10.1371/journal.pone.0243428 (PMC7714355; doi:10.1371/journal.pone.0243428)
Supplement: S2 Fig — (PDF) [file pone.0243428.s004.pdf]

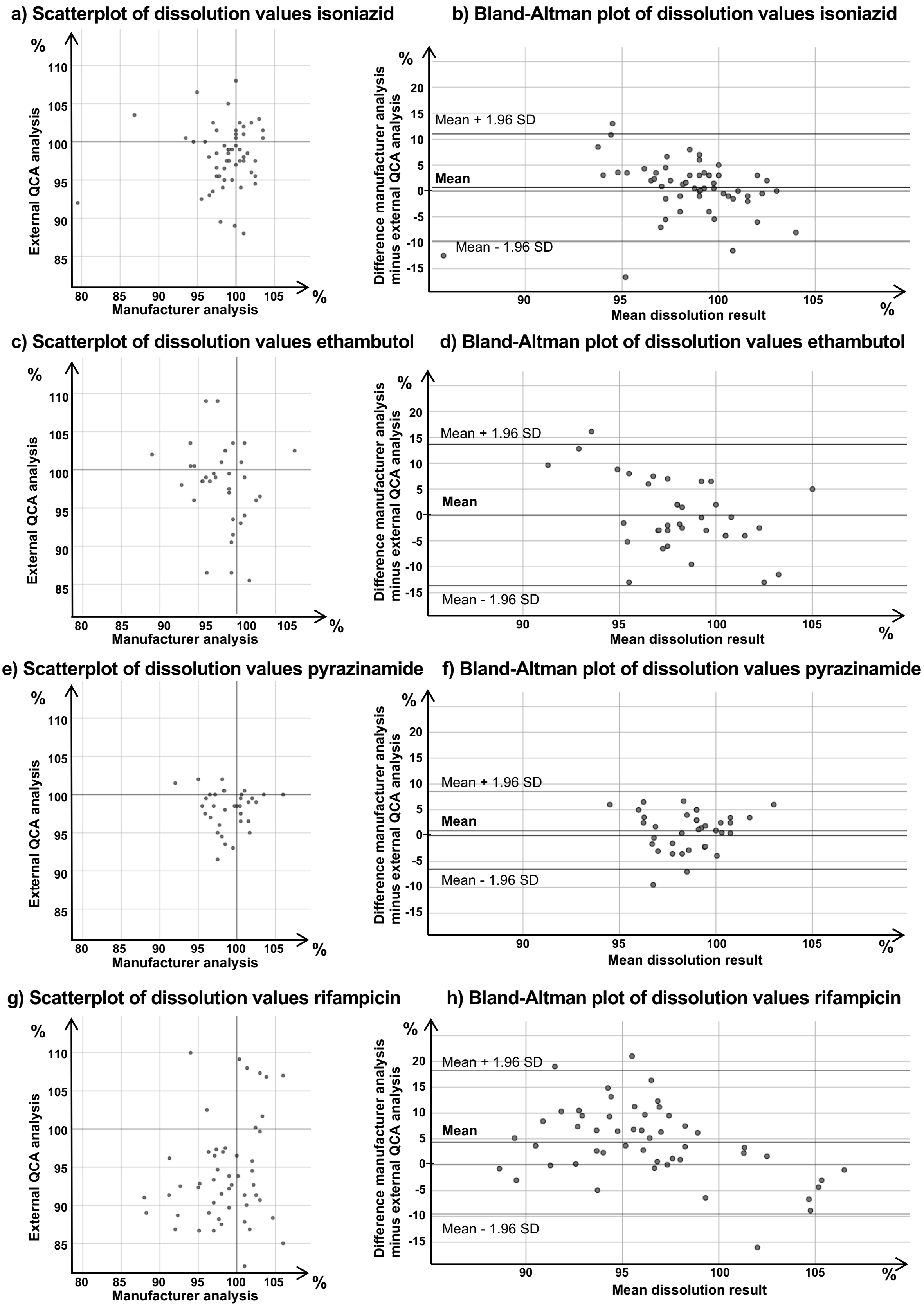

S2 Fig. Inter-laboratory comparison of dissolution results from manufacturer analysis and from external QCA laboratory analysis for the four principal first-line anti-TB agents.
