# Supplementary material for: Quality assurance in anti-tuberculosis drug procurement by the Stop TB Partnership—Global Drug Facility: Procedures, costs, time requirements, and comparison of assay and dissolution results by manufacturers and by external analysis
Source: PLoS One. 2020 Dec 3;15(12):e0243428. doi: 10.1371/journal.pone.0243428 (PMC7714355; doi:10.1371/journal.pone.0243428)
Supplement: S1 Table — (PDF) [file pone.0243428.s005.pdf]

Active Pharmaceutical Ingredient(s)	Strength	Formulation	Packaging	Number of batches 2013-2017	Estimated annual quantity (4/2018-3/2019)	Number of suppliers 2013-2017	Number of suppliers Jan. 2018	Number of WHO prequalified products (Jan. 2018)	Number of SRA- approved products (Jan. 2018)	Number of ERP- approved products (Jan. 2018)	Monograph in Ph. Int. 2017	Monograph in USP 2018	Monograph in BP 2016	Included in WHO EML 2017
**First-line adult and injectable medicines**														
Ethambutol	400 mg	Film coated tablet(s)	Blister(s)	307	30,000,000	4	2	2	0	0	y	y	y	y
Isoniazid	300 mg	Uncoated tablet(s)	Blister(s)	461	200,000,000	2	2	2	0	0	y	y	y	y
Pyrazinamide	400 mg	Uncoated tablet(s)	Blister(s)	217	20,000,000	2	1	1	0	0	y	y	y	y
Pyrazinamide	500 mg	Uncoated tablet(s)	Blister(s)	192	65,000,000	3	2	2	0	0	y	y	y	(n)
Rifabutin	150 mg	Capsule(s)	HDPE jar(s)	12	400,000	2	1	1	0	0	n	y	n (only API)	y
Rifampicin	150 mg	Film coated tablet(s), capsule(s)	Blister(s)	15	50,000	2	1	1	0	0	y	only cap	only cap	y
Rifampicin	300 mg	Film coated tablet(s)	Blister(s)	46	50,000	2	1	0	1	0	y	n (only cap)	y	y
Rifampicin/Isoniazid	150/75 mg	Film coated tablet(s)	Blister(s)	1630	400,000,000	5	3	3	0	0	y	n (only cap)	n (only APIs)	y
Rifampicin/Isoniazid /Ethambutol	150/75/275 mg	Film coated tablet(s)	Blister(s)	447	40,000,000	3	2	2	0	0	y	n (only APIs)	n (only APIs)	y
Rifampicin/Isoniazid /Pyrazinamide/Ethambutol	150/75/400/ 275 mg	Film coated tablet(s)	Blister(s)	3056	250,000,000	4	2	2	0	0	y	y	n (only APIs)	y
Rifapentine	150 mg	Film coated tablet(s)	Blister(s)	2		1	1	0	1	0	n	n (only cap)	n	y (LTBI)
Streptomycin	1 g	Powder for injection	Vial(s)	288	3,000,000	1	2	1	1	0	y	y	y	y (MDR-TB)
**First-line paediatric medicines**														
Ethambutol	100 mg	Film coated tablet(s)	Blister(s)	129	15,000,000	3	2	1	1	0	y	y	y	y
Isoniazid	100 mg	Uncoated tablet(s)	Blister(s)	161	50,000,000	2	3	2	1	0	y	y	y	y
Rifampicin/Isoniazid	75/50 mg	Dispersible tablet(s)	Strip(s)	163	65,000,000	1	1	1	0	0	y	n (only cap)	n (only APIs)	y
Rifampicin/Isoniazid /Pyrazinamide	75/50/150 mg	Dispersible tablet(s)	Blister(s)	87	35,000,000	1	1	1	0	0	y	y	n (only APIs)	y
**Second-line medicines**														
Amikacin	500 mg	Solution for injection	Ampoule(s)	140	500,000	3	2	0	2	0	(n)	y	y	(n) (MDR-TB)
Amoxicillin + Clavulanic acid	250/125 mg	Film coated tablet(s)	Blister(s)	5	*	2	2	0	2	0	n	y	y	n
Amoxicillin + Clavulanic acid	500/125 mg	Film coated tablet(s)	Blister(s)	22	300,000	2	2	0	2	0	n	y	y	n
Amoxicillin + Clavulanic acid	875/125 mg	Film coated tablet(s)	Blister(s)	54	500,000	4	2	0	2	0	n	y	y	n
Amoxicillin + Clavulanic acid	125/31.25 mg/5 mL	Powder for oral suspension	HDPE container(s)	20	*	1	1	0	1	0	n	y	y	n
Capreomycin	0.5 g	Powder for injection	Vial(s)	4	*	1	1	0	0	1	y	y	n	n
Capreomycin	0.75 g	Powder for injection	Vial(s)	16		1	1	0	0	1	y	y	n	n
Capreomycin	1 g	Powder for injection	Vial(s)	312	2,000,000	3	2	1	1	0	y	y	n	y (MDR-TB)
Clarithromycin	250 mg	Film coated tablet(s)	Blister(s)	2		1	0	0	0	0	n	y	y	n
Clarithromycin	500 mg	Film coated tablet(s)	Blister(s)	35		3	0	0	0	0	n	y	y	n
Clofazimine	100 mg	Capsule(s)	HDPE jar(s)	33	12,000,000	1	1	0	1	0	n	y	y	y (MDR-TB)
Cycloserine	250 mg	Capsule(s)	Blister(s)	1098	30,000,000	2	2	2	0	0	y	y	n	y (MDR-TB)
Delamanid	50 mg	Film coated tablet(s)	Blister(s)	19		1	1	0	1	0	n	n	n	y (MDR-TB)
Ethionamide	125 mg	Film coated tablet(s)	Blister(s)	10	210	1	1	1	0	0	n (only API)	y	n (only API)	y (MDR-TB)
Ethionamide	250 mg	Film coated tablet(s)	Blister(s)	408	28,000,000	4	2	2	0	0	n (only API)	y	n (only API)	y (MDR-TB)
Imipenem/Cilastatin	500/500 mg	Solution for injection	Vial(s)	54	250,000	4	3	0	3	0	n	y	n (only APIs)	n
Kanamycin	0.5 g	Powder for injection	Vial(s)	107	*	2	1	1	0	0	y	(n)	n (only API)	(n)
Kanamycin	0.5 g	Solution for injection	Ampoule(s)								(n)	y	n (only API)	(n)
Kanamycin	1 g	Powder for injection	Vial(s)	774	4,500,000	2	2	1	1	0	y	(n)	n (only API)	y (MDR-TB)
Kanamycin	1 g	Solution for injection	Ampoule(s)								(n)	y	n (only API)	(n)
Levofloxacin	250 mg	Film coated tablet(s)	Blister(s)	328	30,000,000	5	2	1	1	0	n	y	n	y (MDR-TB)
Levofloxacin	500 mg	Film coated tablet(s)	Blister(s)	209	1,800,000	4	2	1	1	0	n	y	n	y (MDR-TB)
Linezolid	600 mg	Film coated tablet(s)	Blister(s)	74	55,000,000	3	2	0	2	0	n	n (only API)	n	y (MDR-TB)
Meropenem	1 g	Powder for injection	Vial(s)	2	5,000	1	1	0	1	0	n	y	n (only API)	n
Moxifloxacin	400 mg	Film coated tablet(s)	Blister(s)	210	35,000,000	5	3	2	1	0	n	y	n (only API)	y (MDR-TB)
PAS Acid	4 g	Delayed-release granules	Sachet	136	10,000	1	1	0	1	0	n	n (only API)	n	y (MDR-TB)
PAS Sodium Salt	4 g	Powder for oral solution	Sachet	1874	3,500,000	2	2	2	0	0	n	n (only API)	n	(n)
PAS Sodium Salt	4 g	Delayed-release granules	Sachet, jar								n	n (only API)	n	(n)
Prothionamide	250 mg	Film coated tablet(s)	Blister(s)	217	20,000,000	3	2	2	0	0	n (only API)	n	n	y (MDR-TB, alternative to ethionamide)
Terizidone	250 mg	Capsule(s)	Blister(s)	14	*	1	1	0	1	0	n	n	n	y (MDR-TB, alternative to cycloserine)
**Products not included in GDF Product List April 2018**														
Clofazimine	50 mg	Capsule(s)		13	153,300	1	0	0	0	0	n	y	y	y (MDR-TB)
Ethambutol/Isoniazid	400/150 mg	Film coated tablet(s)		9		1	0	0	0	0	y	n	n	y
Ethambutol	800 mg	Tablet(s)		217		1	0	0	0	0	y	y	y	(n)
Pyrazinamide	750 mg	Tablet(s)		47		2	0	0	0	0	y	y	y	(n)
Rifampicin/Isoniazid	60/30 mg	Dispersible tablet(s)		98		2	0	0	0	0	y	n (only cap)	n (only APIs)	(n)
Rifampicin/Isoniazid	60/60 mg	Dispersible tablet(s)		111		1	0	0	0	0	y	n (only cap)	n (only APIs)	(n)
Rifampicin/Isoniazid /Pyrazinamide	60/30/150 mg	Dispersible tablet(s)		103		2	0	0	0	0	y	y	n (only APIs)	(n)
Rifampicin/Isoniazid	150/150 mg	Film coated tablet(s)		120		1	1	1	0	0	y	n (only cap)	n (only APIs)	y

S1 Table. Details of anti-tuberculosis medicines monitored in the quality control procedure of the Global Drug Facility in the study period 2013-2017.

Sources: GDF-Product List April 2018, GDF FLD and SLD list JAN 2018, Annex H: Indicative non-binding estimated quantities FLD and SLD; 1st April 2018 to 31 March 2019.[20 21] If the product is not mentioned in Annex H (Indicative non-binding estimated quantities FLD and SLD), no number of products is indicated in the column "Estimated annual quantity (4/2018-3/2019)". *) According to Annex H:[20 21] Insufficient demand information to generate volume estimates

Abbreviations: API, Active pharmaceutical ingredient; BP, British Pharmacopoeia; Cap, Capsules; EML, Essential Medicine List; FDC, Fixed Dose Combination; HDPE, High Density Polyethylene; Ph. Int., International Pharmacopoeia; LTBI, Latent TB infection; MDR, Multi drug resistant; n, No (not included in pharmacopeia or EML); (n), different formulation or strength is included in pharmacopeia or EML; PAS, *para*-aminosalicylic acid; USP, United States Pharmacopeia; y, Yes (included in pharmacopeia or EML)
