# Supplementary material for: Quality assurance in anti-tuberculosis drug procurement by the Stop TB Partnership—Global Drug Facility: Procedures, costs, time requirements, and comparison of assay and dissolution results by manufacturers and by external analysis
Source: PLoS One. 2020 Dec 3;15(12):e0243428. doi: 10.1371/journal.pone.0243428 (PMC7714355; doi:10.1371/journal.pone.0243428)
Supplement: S2 Table — (PDF) [file pone.0243428.s006.pdf]

	Assay		Dissolution		Difference	
	**Manufacturer analysis**	**External QCA analysis**	**Manufacturer analysis**	**External QCA analysis**	**Assay**	**Dissolution**
Capreomycin inj.						
N	10	10	-	-	10	-
Min/Max	95.5/107.1%	95.8/109.6%			-6.00/5.40%	
Mean	101.5%	103.0%	-	-	-1.49%	-
Median	101.2%	102.8%	-	-	-0.95%	-
Standard deviation	3.02%	3.77%	-	-	3.27%	-
Cycloserine						
N	10	10	10	10	10	10
Min/Max	97.75/103.1%	92.5/100.8%	95.7/100.5%	89.5/101.5%	-2.05/10.35%	-4.00/6.18%
Mean	100.3%	98.0%	97.9%	96.4%	2.28%	1.55%
Median	100.7%	99.1%	97.5%	97.3%	1.10%	1.92%
Standard deviation	1.87%	2.99%	1.53%	3.31%	4.17%	2.72%
Ethambutol						
N	37	37	37	36	37	36
Min/Max	95.2/104.5%	93.3/106.1%	89.0/107.5%	85.5/109.0%	-7.80/9.70%	-13.00/16.12%
Mean	99.3%	98.6%	98.0%	98.1%	0.71%	0.04%
Median	99.3%	98.4%	98.5%	98.8%	0.30%	-1.66%
Standard deviation	1.55%	3.20%	3.38%	5.45%	3.85%	6.96%
Ethionamide						
N	20	20	20	20	20	20
Min/Max	97.5/104.0%	96.5/104.5%	94.0/102.5%	94.0/104.5%	-5.50/2.80%	-7.50/3.50%
Mean	98.8%	97.2%	99.0%	99.9%	-1.00%	-0.91%
Median	99.0%	96.8%	99.5%	98.5%	-0.71%	-1.00%
Standard deviation	1.45%	2.26%	2.07%	2.65%	2.49%	2.91%
Isoniazid						
N	57	57	57	57	57	57
Min/Max	98.1/104.0%	90.9/106.1%	79.5/103.5%	88.0/108.0%	-9.80/9.60%	-16.65/13.00%
Mean	98.8%	97.2%	98.8%	98.1%	1.60%	0.67%
Median	99.0%	96.8%	99.5%	98.5%	2.10%	1.49%
Standard deviation	1.63%	3.11%	3.80%	4.01%	3.54%	5.28%
Kanamycin inj.						
N	15	15	-	-	15	-
Min/Max	96.9/106.1%	90.0/106.1%	-	-	-15.20/15.30%	-
Mean	100.9%	101.7%	-	-	-0.79%	-
Median	101.0%	100.3%	-	-	0.02%	-
Standard deviation	2.69%	6.59%	-	-	8.67%	-
Levofloxacin						
N	15	15	15	15	15	15
Min/Max	98.0/102.5%	96.7/104.2%	96.4/103.5%	98.5/104.0%	-4.10/2.90%	-7.60/3.00%
Mean	100.3%	100.8%	99.7%	101.2%	-0.54%	-1.77%
Median	100.0%	100.7%	99.0%	101.0%	0.00%	-1.35%
Standard deviation	1.50%	1.86%	2.05%	1.59%	2.19%	2.75%
Linezolid						
N	9	9	9	9	9	9
Min/Max	95.0/102.3%	99.0/102.3%	94.0/102.5%	95.0/101.0%	-4.80/3.30%	-6.50/6.00%
Mean	99.5%	100.5%	97.1%	98.5%	-1.9%	-1.44%
Median	100.3%	100.3%	96.0%	99.5%	-0.70%	-0.50%
Standard deviation	2.35%	1.06%	2.95%	2.22%	2.56%	4.14%
Moxifloxacin						
N	12	12	12	12	12	12
Min/Max	97.9/103.7%	96.2/103.3%	96.0/100.1%	97.0/102.5%	-2.60/6.00%	-4.05/3.05%
Mean	100.7%	100.0%	98.5%	99.2%	0.66%	-0.66%
Median	100.6%	100.2%	99.0%	98.8%	-0.10%	-1.00%
Standard deviation	1.81%	1.92%	1.42%	1.86%	2.97%	2.24%
Para-aminosalicylic acid						
N	5	5	5	5	5	5
Min/Max	98.7/102.5%	95.9/100.3%	85.5/91.0%	89.5/97.5%	0.20/6.60%	-7.50/-1.00%
Mean	100.2%	98.1%	88.4%	93.8%	2.10%	-5.40%
Median	99.5%	98.7%	88.5%	93.5%	0.90%	-6.50%
Standard deviation	1.60%	1.92%	1.95%	2.99%	2.66%	2.63%
Protionamide						
N	10	10	10	10	10	10
Min/Max	99.0/100.9%	97.2/101.9%	96.6/103.5%	96.0/101.15%	-2.60/2.80%	-2.90/4.00%
Mean	99.6%	99.3%	99.7%	99.1%	0.31%	0.62%
Median	99.5%	99.5%	98.8%	99.5%	0.10%	1.28%
Standard deviation	0.60%	1.50%	2.14%	1.55%	1.68%	2.45%
Pyrazinamide						
N	37	37	36	37	37	37
Min/Max	96.6/101.4%	95.2/101.9%	92.0/106.0%	91.5/102.0%	-3.50/5.45%	-9.50/6.67%
Mean	99.6%	98.8%	99.1%	98.0%	077%	1.00%
Median	99.5%	99.2%	99.0%	99.0%	0.44%	1.62%
Standard deviation	1.08%	1.46%	2.72%	2.51%	1.96%	3.82%
Rifampicin						
N	51	51	51	51	51	51
Min/Max	96.4/108.2%	93.3/105.4%	88.0/106.0%	82.0/110.0%	-8.60/9.60%	-16.00/21.00%
Mean	100.1%	98.8%	98.5%	94.1%	1.30%	4.39%
Median	100.1%	98.6%	99.0%	92.67%	1.60%	3.67%
Standard deviation	1.95%	3.05%	4.25%	6.67%	3.60%	7.09%

S2 Table. Descriptive summary of assay and dissolution data for all 13 active pharmaceutical ingredients which had been analysed in the study period by the external QCA.
