# Supplementary material for: Quality assurance in anti-tuberculosis drug procurement by the Stop TB Partnership—Global Drug Facility: Procedures, costs, time requirements, and comparison of assay and dissolution results by manufacturers and by external analysis
Source: PLoS One. 2020 Dec 3;15(12):e0243428. doi: 10.1371/journal.pone.0243428 (PMC7714355; doi:10.1371/journal.pone.0243428)
Supplement: S3 Table — (PDF) [file pone.0243428.s007.pdf]

**Data set compared (Manufacturer analysis minus external QCA analysis)**	**Difference Mean (95 % CI)**	**Difference Standard deviation**	**Correlation coefficient (P value)**	**Limits of agreement (95 % CI)**
Assay (n= 288)	0.67 % (0.24 to 1.10 %)	3.74 %	0.035 (p=0.559)	- 6.69 % (- 7.44 to - 5.94%)<br>+ 8.03 % (7.28 to 8.78 %)
Dissolution (n= 261)	0.88 % (0.20 to 1.56 %)	5.55 %	0.132 (p=0.034)	- 10.05 % (- 11.22 to - 8.88 %)<br>+ 11.81 % (10.64 to 12.98 %)
Isoniazid assay (n= 57)	1.60 % (0.66 to 2.54 %)	3.54 %	-0.018 (p=0.894)	- 5.49 % (- 7.12 to - 3.86 %)<br>+ 8.69 % (7.06 to 10.32 %)
Ethambutol assay (n= 37)	0.71 % (- 0.57 to 1.99 %)	3.85 %	- 0.221 (p=0.188)	- 7.10 % (- 9.32 to – 4.87 %)<br>+ 8.52 % (6.29 to 10.74 %)
Pyrazinamide assay (n= 37)	0.77 % (0.12 to 1.42 %)	1.96 %	- 0.173 (p=0.305)	- 3.21 % (- 4.34 to - 2.07 %)<br>+ 4.75 % (3.61 to 5.88 %)
Rifampicin assay (n= 51)	1.30 % (0.29 to 2.31 %)	3.60 %	0.010 (p=0.945)	- 5.93 % (- 7.68 to - 4.18 %)<br>+ 8.53 % (6.78 to 10.28 %)
Isoniazid dissolution (n= 57)	0.67 % (- 0.73 to 2.07 %)	5.28 %	0.088 (p=0.516)	- 9.91 % (- 12.33 to - 7.48 %)<br>+ 11.25 % (8.82 to 13.67 %)
Ethambutol dissolution (n= 36)	0.04 % (- 2.28 to 2.36 %)	6.96 %	- 0.201 (p=0.239)	- 14.08 % (- 18.09 to - 10.06 %)<br>+ 14.16 % (10.14 to 18.17 %)
Pyrazinamide dissolution (n= 36)	1.00 % (- 0.27 to 2.27 %)	3.82 %	- 0.056 (p=0.747)	- 6.75 % (- 8.95 to - 4.54 %)<br>+ 8.75 % (6.54 to 10.95 %)
Rifampicin dissolution (n= 51)	**4.39 %** (2.40 to 6.38 %)	7.09 %	0.217 (p=0.127)	- 9.85 % (- 13.30 to - 6.40 %)<br>+ 18.63 % (15.18 to 22.08 %)
Kanamycin assay (n=81) [a]	- 0.65 % (- 2.19 to 0.89 %)	6.97 %	- 0.063 (p=0.579)	- 14.52 % (- 17.19 to - 11.85 %)<br>+ 13.22 % (10.55 to 15.89 %)
Kanamycin assay, manufacturer 1 (n=55) [a]	- 0.19 % (- 2.19 to 1.81 %)	7.40 %	- 0.159 (p=0.246)	- 15.03 % (- 18.49 to - 11.56 %)<br>+ 14.65 % (11.18 to 18.11 %)
Kanamycin assay, manufacturer 2 (n=16) [a]	1.39 % (0.65 to 2.13 %)	1.38 %	0.355 (p=0.177)	- 1.55 % (- 2.83 to - 0.28 %)<br>+ 4.33 % (3.06 to 5.61 %)

S3 Table. Detailed results of Bland-Altman analysis

[a] Originally 15 kanamycin injection samples had been selected for the inter-laboratory comparison of results (see Methods section); a summary of these data is included in Table S2. Subsequently, the data of all 81 kanamycin injection samples which had been analysed in the study period by the external QCA were investigated; a summary of these data is given in Table S4.
The highest observed bias is highlighted in bold print.
