# Supplementary material for: Quality assurance in anti-tuberculosis drug procurement by the Stop TB Partnership—Global Drug Facility: Procedures, costs, time requirements, and comparison of assay and dissolution results by manufacturers and by external analysis
Source: PLoS One. 2020 Dec 3;15(12):e0243428. doi: 10.1371/journal.pone.0243428 (PMC7714355; doi:10.1371/journal.pone.0243428)
Supplement: S4 Table — In addition, data from the two main manufacturers supplying kanamycin are presented. (PDF) [file pone.0243428.s008.pdf]

	Assay		
	Manufacturer analysis	External QCA analysis	Difference
All kanamycin samples			
N	81	81	81
Mean	101.8 %	102.5 %	-0.65 %
Median	101.5 %	101.7 %	-0.20 %
Standard deviation	2.45 %	6.25 %	6.97 %
Kanamycin samples from manufacturer 1			
N	55	55	55
Mean	102.8 %	103.0 %	-0.19 %
Median	103.0 %	104.1 %	0.50 %
Standard deviation	2.2 %	6.78 %	7.40 %
Kanamycin samples from manufacturer 2			
N	16	16	16
Mean	101.0 %	99.6 %	1.39 %
Median	101.0 %	99.8 %	1.07 %
Standard deviation	0.37 %	1.48 %	1.38 %

S4 Table. Descriptive summary of assay data for all 81 kanamycin injection samples which had been analysed in the study period by the external QCA. In addition, data from the two main manufacturers supplying kanamycin are presented.
