# Supplementary material for: Quality assurance in anti-tuberculosis drug procurement by the Stop TB Partnership—Global Drug Facility: Procedures, costs, time requirements, and comparison of assay and dissolution results by manufacturers and by external analysis
Source: PLoS One. 2020 Dec 3;15(12):e0243428. doi: 10.1371/journal.pone.0243428 (PMC7714355; doi:10.1371/journal.pone.0243428)
Supplement: S5 Table — (PDF) [file pone.0243428.s009.pdf]

		USP 2018	Ph. Int. 2017
Rifampicin capsules and tablets	Medium, volume<br>Apparatus:<br>rpm, time:<br>Limit:	0.1N HCl, 900mL<br>1<br>100 rpm, 45min<br>>75% **(only for capsules)**	Buffer pH 6.8, 0.25% SDS TS, 500mL<br>2<br>75 rpm, 30min<br>>80%
Rifampicin and isoniazid tablets	Medium, volume<br>Apparatus:<br>rpm, time:<br>Limit:	0.1N HCl, 900mL<br>1<br>100 rpm, 45min<br>>75% **(only for capsules)**	"Dissolution test. [To be added for rifampicin]"<br>(**for dispersible tablets**; for tablets not mentioned)
Rifampicin/ isoniazid/ethambutol tablets	Medium, volume<br>Apparatus:<br>rpm, time:<br>Limit:	No monograph	"Dissolution test. [To be added for rifampicin]"
Rifampicin/ isoniazid/pyrazin-amide tablets	Medium, volume<br>Apparatus:<br>rpm, time:<br>Limit:	Simulated gastric fluid TS without pepsin, 900mL<br>1<br>100rpm, 30min<br>>80%	"Dissolution test. [To be added for rifampicin]"<br>(**for dispersible tablets**; for tablets not mentioned)
Rifampicin/isoniazid/ pyrazinamide/ ethambutol tablets	Medium, volume<br>Apparatus:<br>rpm, time:<br>Limit:	10mM Sodium phosphate buffer pH 6.8, 900mL<br>2<br>100rpm, 45min<br>>75%	Not mentioned

S5 Table. Dissolution testing conditions for solid oral formulations containing rifampicin in the United States Pharmacopeia 2018 and the International Pharmacopoeia 2017. (TS=Test Solution)
